# Changes in quality of life 6 months after parathyroidectomy for primary hyperparathyroidism

**DOI:** 10.1530/EC-21-0630

**Published:** 2022-02-23

**Authors:** Julie Wulf Christensen, Karin Folmer Thøgersen, Lars Thorbjørn Jensen, Martin Krakauer, Bent Kristensen, Finn Noe Bennedbæk, Bo Zerahn

**Affiliations:** 1 Department of Nuclear Medicine, Herlev and Gentofte Hospital, Herlev, Denmark; 2 Department of Clinical Physiology and Nuclear Medicine, Bispebjerg and Frederiksberg Hospital, Copenhagen, Denmark; 3 Division of Endocrinology, Department of Medicine, Herlev and Gentofte Hospital, Herlev, Denmark

**Keywords:** quality of life, hyperparathyroidism, SF-36, parathyroidectomy

## Abstract

**Objective:**

The extent of symptoms due to primary hyperparathyroidism (PHPT) depends on the population being studied. PHPT is mainly discovered incidentally through routine laboratory findings. Less is known about patient-experienced improvement following successful parathyroidectomy. The aim of our study was to assess the changes in the quality of life (QoL) after successful surgery using an SF-36 questionnaire.

**Design:**

This is a prospective cohort study based on questionnaires.

**Methods:**

Forty consecutive patients diagnosed with PHPT were prospectively administered an SF-36 questionnaire before and 6 months after successful parathyroidectomy. A subgroup of 18 patients answered the questionnaire at 1 and 3 months after surgery. Successful surgery was based on biochemistry and pathology reports as confirmed by an endocrinologist. Results of each SF-36 subcategory were compared to the results at baseline in order to detect changes in patient-reported QoL after successful surgery.

**Results:**

There were significant improvements in six of eight SF-36 subcategories: vitality (*P* = 0.0001), physical functioning (*P* = 0.04), general health perception (*P* = 0.004), physical role functioning (*P* = 0.04), social role functioning (P = 0.004), and mental health perception (*P* = 0.0001). Changes appeared within a month after surgery with no further significant changes at later time points.

**Conclusions:**

Parathyroidectomy significantly improves QoL as measured by a decrease in SF-36 scores as early as 1 month after successful parathyroidectomy. The SF-36 QoL questionnaire is suitable for monitoring changes in patient well-being after successful parathyroidectomy.

## Introduction

Primary hyperparathyroidism (PHPT) is characterised by hyperfunctioning tissue in one or more of the parathyroid glands, causing an increase in parathyroid hormone (PTH). This results in renal tubular reabsorption and osteoclastic bone resorption. Moreover, increased PTH indirectly results in increased intestinal Ca^2+^ absorption promoted by an increased renal activation of vitamin D (1, 2, 3).

The only curative treatment is parathyroidectomy (PTx), that is the surgical removal of the hyperfunctioning parathyroid gland(s) (HPG(s)), preceded by imaging in order to offer a minimally invasive procedure. In previous publications, we have evaluated imaging modalities for this purpose (4, 5).

The clinical picture of PHPT has changed over the last decades, mainly due to the early detection of hypercalcemia and often occurs as an asymptomatic or a mildly symptomatic disease. Symptoms of PHPT are diverse and often non-specific and include musculoskeletal symptoms (decreased muscle strength and painful joints), neuropsychiatric symptoms (e.g. anxiety, depression/mood change, fatigue, and memory problems), and gastrointestinal discomfort, including constipation (1, 2). All of these factors may potentially affect the quality of life (QoL).

To easily evaluate self-reported QoL, RAND Health Care (Santa Monica, CA, USA) has developed a 36-item short form health survey (SF-36) (6). The SF-36 questionnaire comprises 36 questions divided into 8 sections. The score from each section is directly transformed into a scale of 0–100 (the lower the score, the more disability; the higher the score, the less disability). Scoring is done according to a standard scoring algorithm (6, 7). Questions are divided into the following sections: vitality, physical functioning, bodily pain, general health perceptions, physical role functioning, emotional role functioning, social role functioning, and mental health.

The SF-36 questionnaire has previously been used to evaluate a wide array of different populations, including older urban adults, surgical nurses, or middle-aged Swedish general population, or to evaluate the status during certain diseases or following treatment (i.e. after burn injuries or hip arthroplasty) (8, 9, 10).

The neuropsychiatric effects (e.g. depression and anxiety) of PHPT have been examined using various questionnaires and tests before PTx and up to 10 years later. In 2002, Pasieka *et al.* found significant alleviation of disease-specific symptoms as well as improvement in QoL 12 months after PTx as compared to preoperative conditions (11). Symptoms and QoL remained better after 10 years follow-up (12). Several publications have described a higher proportion of patients with neuropsychological symptoms before PTx as compared to control groups without PHPT as well as significant decrease in the proportion of patients with depression or anxiety after PTx (9, 13, 14). Follow-up periods range from 19 days to 12 months after PTx. Additionally, Weber *et al.* found a higher prevalence of depression in patients with higher baseline Ca^2+^ but no correlation to age and gender (13). The number of patients included in these studies ranged from 39 to 202.

Webb *et al.* have developed a disease-specific QoL questionnaire (PHPQoL) containing 16 questions that focus on emotional and physical domains (15). This has been used alongside the SF-36 questionnaire with similar results, that is a significant improvement in scores up to 12 months after PTx (16, 17, 18).

Findings differ on whether impaired QoL is associated with degree of hypercalcaemia, that is higher preoperative Ca^2+^ is associated with lower baseline scores. Ejlsmark-Svensson *et al.* found an association, while Pasieka *et al.* did not (11, 18).

In the present study, we intend to focus on the effects on overall patient-reported QoL up to 6 months after successful PTx and to determine whether changes could already be registered after 1 and/or 3 months.

For this purpose, we report if and when the eight categories of SF-36 improve after surgery and whether or not changes are affected by gender, age, or preoperative calcium level to assist in the evaluation of patients with PHPT in a clinical setting.

## Materials and methods

### Patient inclusion and treatment

All patients were diagnosed with PHPT at the Department of Medicine, Division of Endocrinology, Herlev and Gentofte Hospital, Denmark, and were included in a previous study about imaging prior to PTx. Details on inclusion criteria and results are outlined in a previous publication (5). From September 2019 to December 2020, 62 consecutive patients included in these studies were also invited to participate in the present study, 47 of whom were accepted.

**Figure 1:**
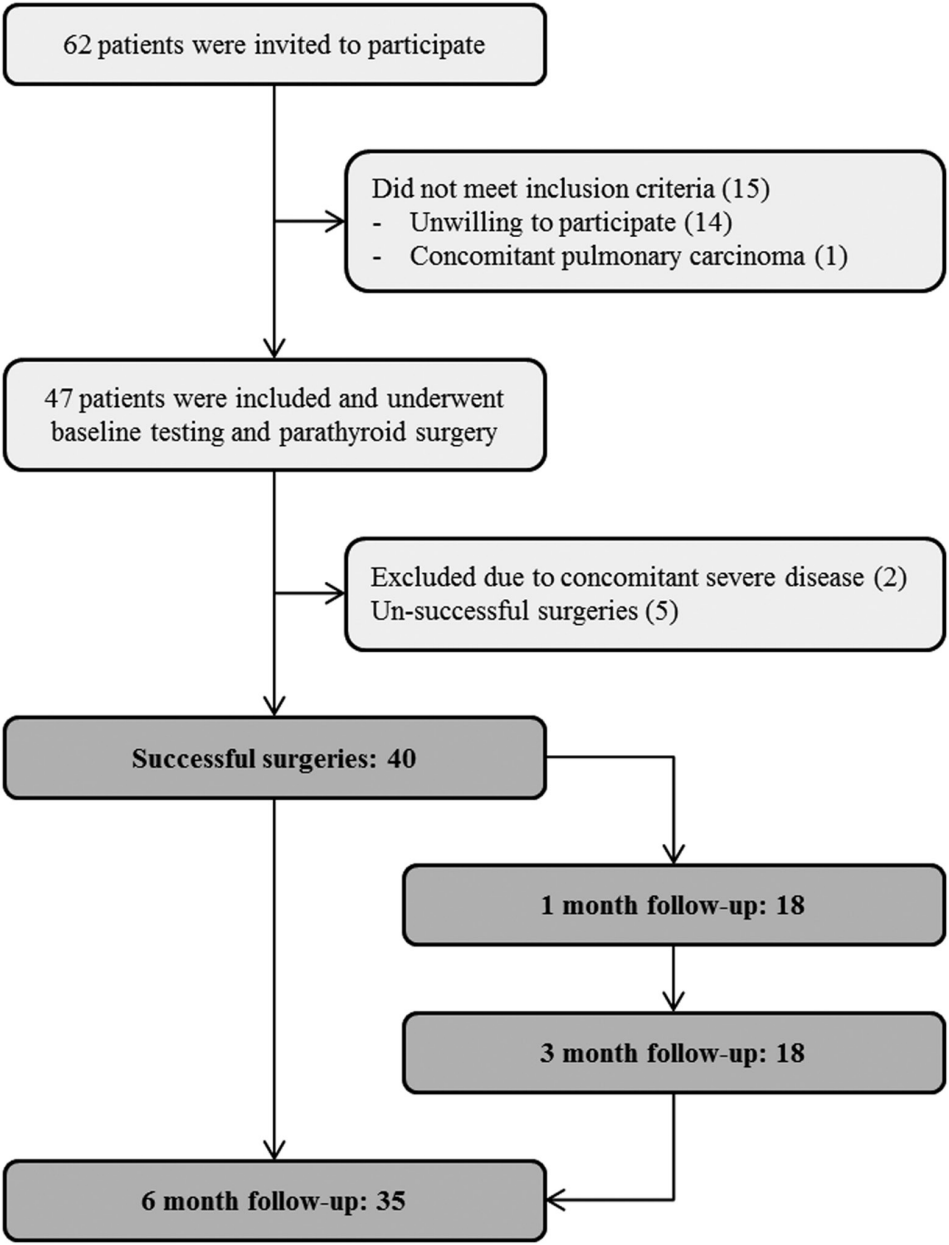
Inclusion flow. At 1 and 3 months follow-up, 20 patients were invited and all showed up. At 6 months follow-up, all patients were invited. Four patients cancelled and one had died from causes unrelated to hyperparathyroidism.

Figure 1 shows the inclusion flow diagram. All included patients responding to the SF-36 questionnaire had undergone routine PTx with the removal of the suspected HPG(s). Successful surgery was confirmed by an intraoperative decrease in PTH of ≥50% and/or to within normal range, pathology confirmation of hyperfunctioning parathyroid tissue and postoperative normalisation of Ca^2+^ and PTH.

All patients completed the QoL questionnaire and underwent blood testing before curative intended PTx, as well as 6 months after surgery. A subset of patients was further invited for testing 1 and 3 months after PTx in order to detect any early changes. A timeframe of up to 3 months was deemed acceptable between baseline tests and PTx. In cases where this was exceeded due to postponed PTx, new baseline tests were performed and the previous results were discarded.

Patients with significant comorbidities who were diagnosed or progressing during the trial (e.g. hip surgery, cardio- or cerebrovascular disease including stroke (apoplexy), or cancer) were excluded as these would likely alter the outcome of the QoL assessment since these competing conditions may have affected the QoL level attributed to PHPT alone.

### Quality of life

Within 2 weeks of each visit, a previously validated Danish translation of the SF-36 v.1 questionnaire was sent to each patient via the secure web application REDCap™ (8, 19). Patients were given the option to respond online prior to their visit or on paper during the visit. In the latter case, patients were given the time and space to complete the questionnaire without interference.

Results were used to calculate the scores in the categories vitality, physical functioning, bodily pain, general health perceptions, physical role functioning, emotional role functioning, social role functioning, and mental health according to the algorithm provided by RAND (7).

### Statistical analysis

Due to the unbalanced data with correlated observations, the data were analysed in repeated measures in time random effects design using linear mixed models (LMM) to avoid too small standard errors and to make the results usable in other populations.

Differences between individual measurement times were assessed by marginal means for each LMM with correction for pairwise comparisons using Tukey adjustments.

All statistical calculations were done using the statistical software R, version 4.0.2, June 2020, (https://www.R-project.org/), and the following packages: *stats, tidyverse, ggpubr, rstatix, nlme, emmeans*, and *readxl*.

Symptoms reported by PHPT patients are heterogeneous, and while some patients are severely affected, others may be almost asymptomatic and have no room for improvement. To assess the changes in the former group, we added the baseline value of each subcategory as a covariate in the LMM. To compare those who were more affected at baseline with those less affected or unaffected, we also subcategorised patients into two groups: patients with baseline values in the lower-third compared to baseline in the upper two-thirds. This was done separately for each SF-36 category.

Furthermore, we adjusted the LMM for inclusion pre- and post-COVID-19 lockdown, gender, age, and preoperative Ca^2+^ to assess possible differences in QoL evolution.

The study was conducted in accordance with the Helsinki 2 declaration and the International Council for Harmonisation Guideline for Good Clinical Practice and approved by the Research Ethics Committee of the Capital Region of Denmark (Journal-no: H-18012490, date of approval: 18 June 2018). Written consent has been obtained from each patient after a full explanation of the purpose and nature of all procedures used.

## Results

### Patient characteristics

A total of 47 patients met the inclusion criteria and were included in the present study. Of these, 42 (89%) had a successful operation based on follow-up biochemistry and chart notes from the treating physician in the Department of Medicine, Division of Endocrinology. Patients were predominantly female (62%) with a median age of 63 years at inclusion (range: 28–82 years). After 6 months, 41 patients participated, three declined testing and one died of other causes before 6 months had passed. Only 20 patients were invited to participate at 1 and 3 months follow-up, of which 18 surgeries (90%) were successful. The gender distribution was similar at all follow-ups. See Table 1 for details.

**Table 1:** Baseline characteristics of the 40 patients with successful PTx and no severe and progressing concurrent disease.

	*n*
Number of patients	40
Gender	
Female	26 (65 %)
Male	14 (35 %)
	**Median** (range)
Age at inclusion (years)	62 (28–82)
Height (cm)	172 (147–190)
Weight (kg)	77 (49–133)
BMI (kg/m^2^)	26 (18–43)
Preoperative PTH (pmol/L)	14.7 (4.9–36.3)
Preoperative Ca^2+^ (mmol/L)	1.46 (1.35–1.71)
Days from baseline to PTx	12 (1–55)

Ca^2+^, ionised calcium, reference: 1.18–1.32 mmol/L; PTx, parathyroidectomy; PTH, parathyroid hormone, reference: 2.0–8.5 pmol/L.

A 75-year-old male patient had concomitant severe and increasing heart failure, which significantly affected his physical and mental condition. Further, a 65-year-old male patient had deteriorating general health unrelated to PHPT diagnosis. After PTx he was referred to relevant departments for diagnosis and proper treatment. Both patients were excluded from all calculations.

### Quality of life

**Figure 2:**
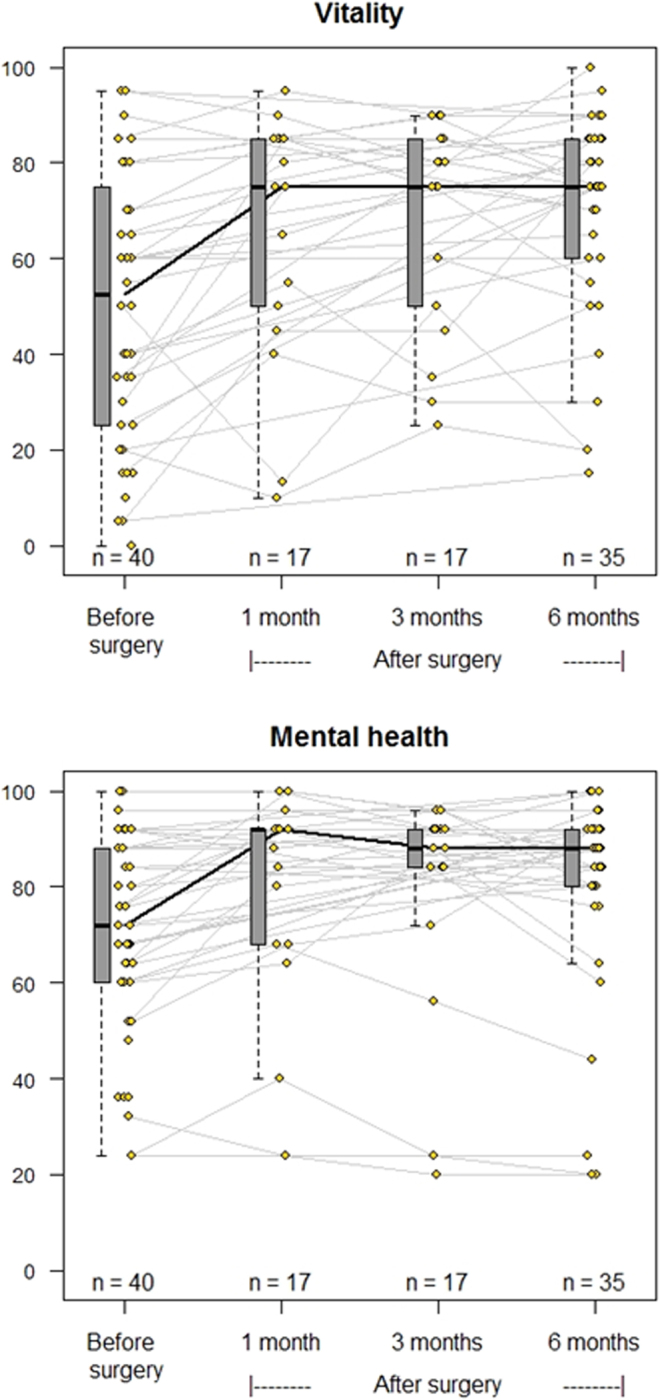
Spaghetti plots of the evolution of quality of life. SF-36 categories vitality (top) and mental health perception (bottom). Grey lines indicate the changes to each individual patient, while a black line marks the change to median values. Change from baseline to 6 months based on estimated marginal means analysis using Tukey adjustments: *P* values < 0.0001.

**Figure 3:**
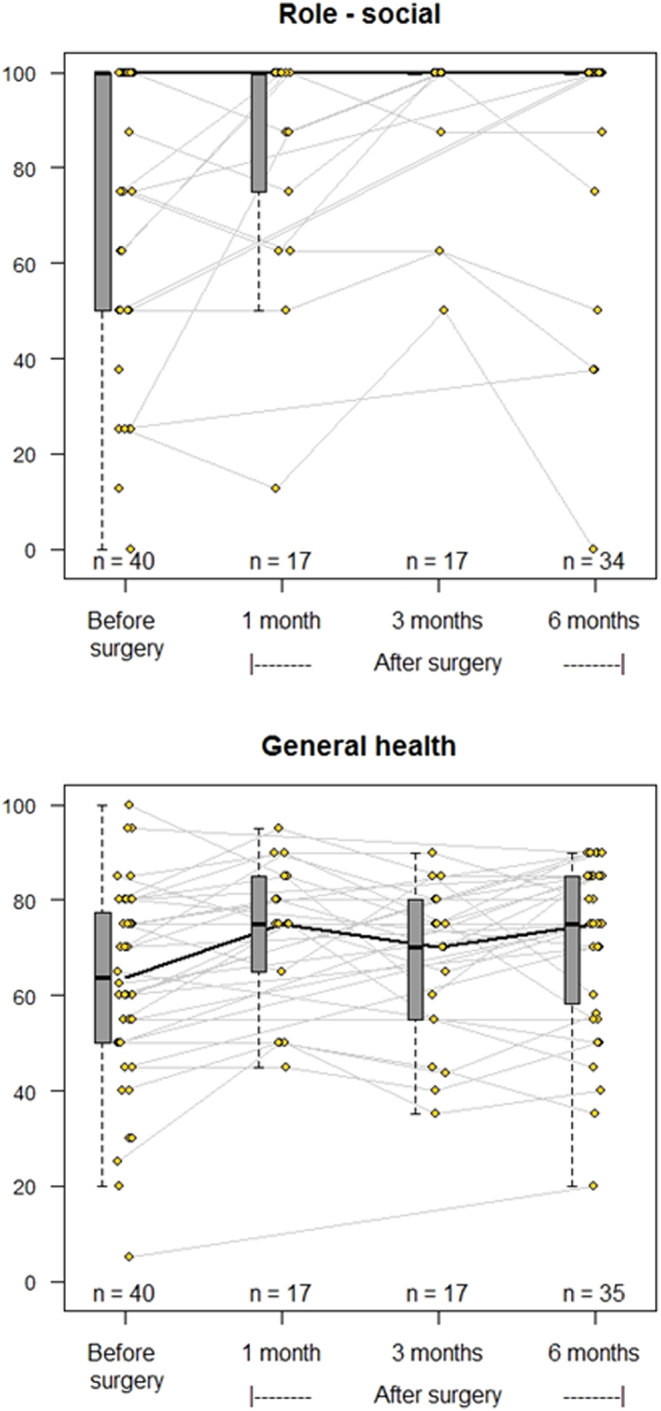
Spaghetti plots of the evolution of quality of life. SF-36 categories social role functioning (top) and general health perception (bottom). Grey lines indicate the changes to each individual patient, while the black line marks the change to median values. Change from baseline to 6 months based on estimated marginal means analysis using Tukey adjustments: *P*values = 0.004.

**Figure 4:**
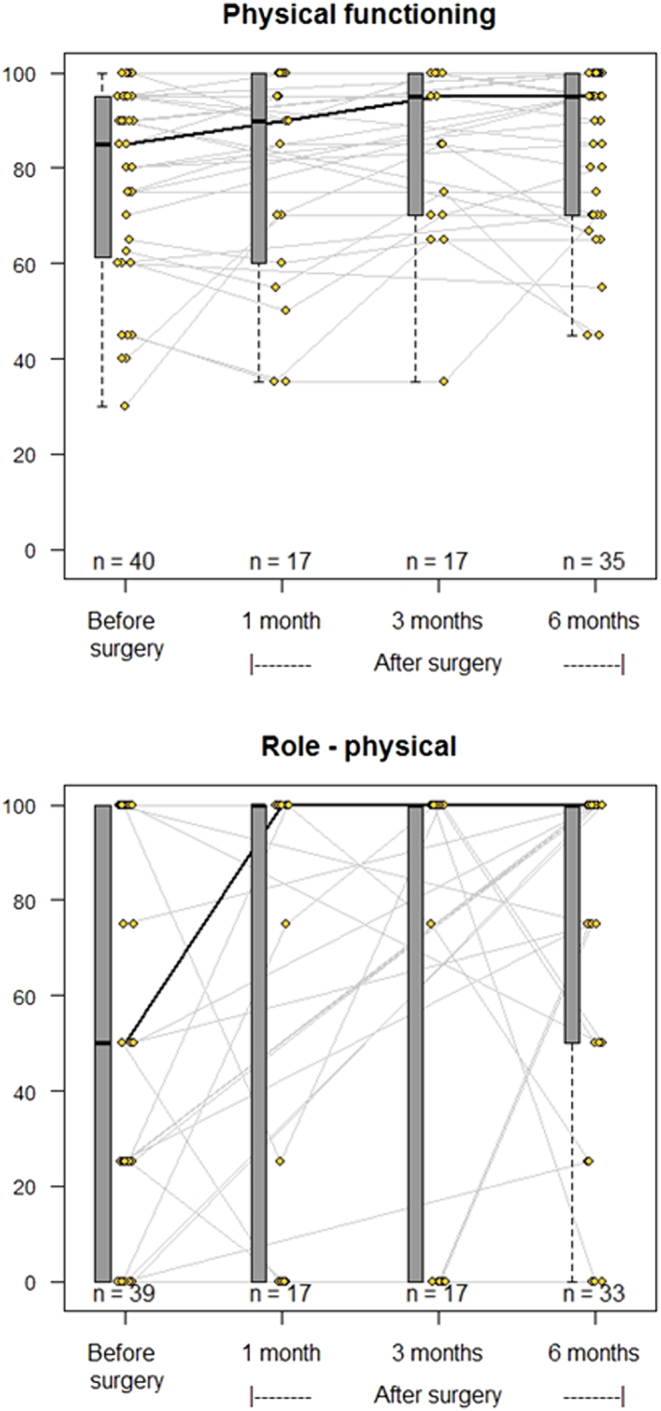
Spaghetti plots of the evolution of quality of life. SF-36 categories Physical functioning (top) and physical role functioning (bottom). Grey lines indicate the changes to each individual patient, while the black line marks the change to median values. Change from baseline to 6 months based on estimated marginal means analysis using Tukey adjustments: *P* values = 0.04.

**Figure 5:**
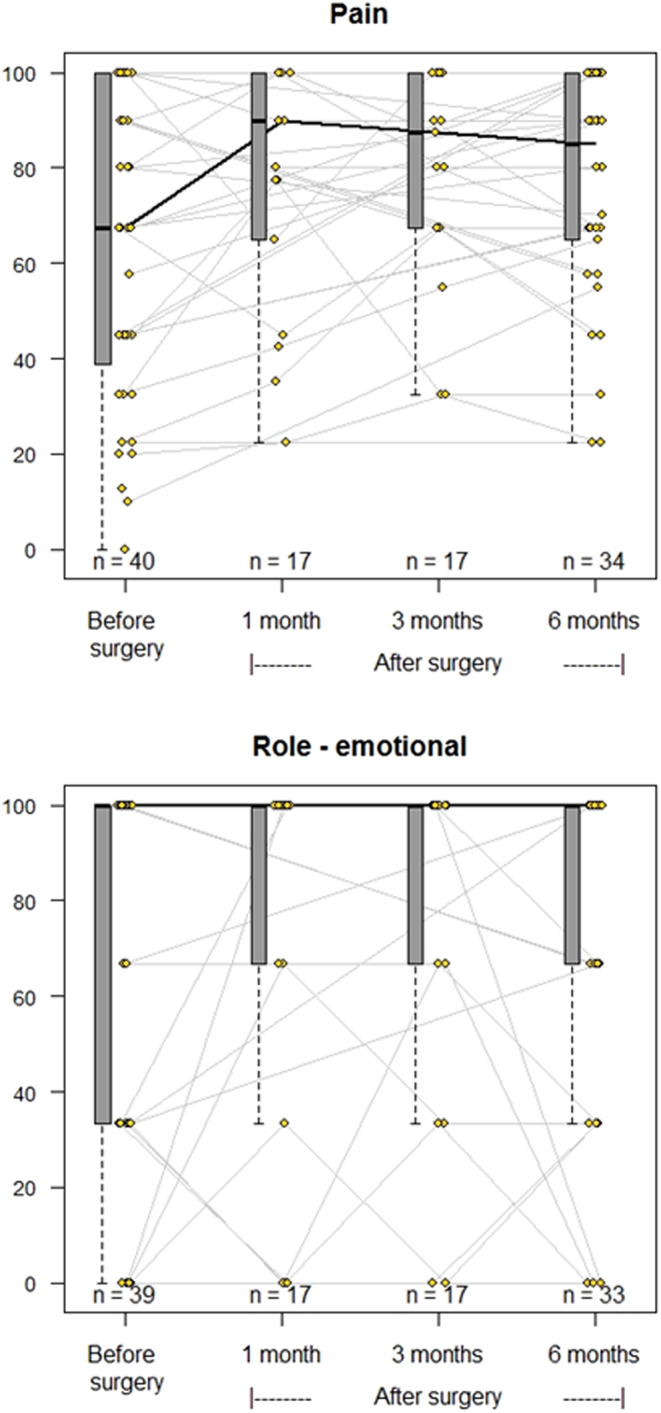
Spaghetti plots of the evolution of quality of life. SF-36 categories Bodily pain (top) and emotional role functioning (bottom). Grey lines indicate the changes to each individual patient, while the black line marks the change to median values. Change from baseline to 6 months based on estimated marginal means analysis using Tukey adjustments: *P* values = NS (0.06 and 0.34, respectively).

The results from QoL before and after curative PTx are displayed in Fig. 2 (vitality and mental health), Fig. 3 (social role functioning and general health perception), Fig. 4 (physical functioning and physical role functioning), and Fig. 5 (bodily pain and emotional role functioning). At baseline, more than 15% of patients had a value of ‘0’ in the physical and emotional role functioning subcategories, while over 15% of patients had a score of ‘100’ in all categories except vitality, general health perception, and mental health perception, limiting the possibility of increase after PTx.

Table 2 displays the median values and 25/75 percentiles at each time point from baseline through 1, 3, and 6 months after successful surgery as well as a *P* value depicting significant overall change. Six of eight subcategories improved significantly within 6 months of successful PTx. There were no significant changes in emotional role functioning or bodily pain within this time frame.

**Table 2:** Results of quality of life questionnaires (successful surgeries only).

**SF-36 category**	Baseline (*n* = 40)	1 month post-PTx (*n* = 18, 38%)	3 months post-PTx (*n* = 18, 38%)	6 months post-PTx (*n* = 35, 77%)	*P* value
	Median (25–75%)	Median (25–75%)	Median (25–75%)	Median (25–75%)	
Vitality	55 (28–75)	75 (50–85)	75 (50–85)	75 (60–85)	0.0001
Physical functioning	85 (64–95)	90 (60–100)	95 (70–100)	95 (70–100)	0.038
Bodily pain	67.5 (45–100)	90 (65–100)	87.5 (68–100)	85 (66–100)	0.056
General health perception	65 (50–78)	75 (65–85)	70 (55–80)	75 (58–85)	0.004
Physical role functioning	50 (0–100)	100 (0–100)	100 (0–100)	100 (50–100)	0.043
Emotional role functioning	100 (33–100)	100 (67–100)	100 (67–100)	100 (42–100)	0.338
Social role functioning	100 (56–100)	100 (75–100)	100 (100–100)	100 (100–100)	0.004
Mental health	72 (60–88)	92 (68–92)	88 (84–92)	88 (80–92)	0.0001

Based on estimated marginal means analysis *P* values < 0.05 indicate significant change within the linear mixed model fitted over all measured time points.

The changes to the median of the six subcategories with significant changes overall are shown in Table 3, with individual significance testing to examine when the change was apparent. With very few exceptions, the 25-percentile change was positive, meaning that changes in QoL were almost always in an upward direction. The subcategories vitality, general health perception, and mental health perception increased significantly as early as 1 month after successful surgery. The increase in these subcategories, as well as both physical and social role functioning, persisted at 6 months.

**Table 3:** Median SF-36 score changes and their 25/75 percentiles from baseline and *P* values – of those with significant overall change only.

SF-36 category	1 month post-PTx Change from baseline		3 months post-PTx Change from baseline		6 months post-PTx Change from baseline	
	Median change (25–75%)	*P* value	Median change (25–75%)	*P* value	Median change (25–75%)	*P*value
Vitality	10 (5–20)	0.006	10 (5–20)	0.001	12.5 (5–20)	<0.0001
Physical functioning	0 (−7.5–5)	0.966	5 (0–10)	0.068	5 (0–10)	0.151
General health perception	5 (0–15)	0.016	0 (−5–5)	0.925	5 (−5–15)	0.021
Physical role functioning	0 (0–0)	0.545	0 (0–0)	0.264	0 (0–50)	0.032
Social role functioning	0 (−12.5–0)	0.344	0 (0–25)	0.006	0 (0–12.5)	0.035
Mental health	8 (0–16)	0.0004	4 (−4–20)	0.002	4 (−3–16)	0.005

#### Gender differences

Incorporating gender as a covariate in the LMM rendered no significant influence by gender in any subcategory (*P*values > 0.38).

We then separated men and women into two groups and repeated the above calculations for each gender separately using the *emmeans* package in R. The previously observed improvement in vitality score was only significant in women (*P* values were 0.002, 0.002, and 0.0001 after 1, 3, and 6 months, respectively), while the mental health perception score improved for women as early as 1 month after successful PTx (*P* = 0.002) and for men after 6 months (*P* = 0.046) despite similar baseline values. Social role functioning improved significantly among men after 3 months (*P* = 0.016), but the improvement was not sustained at 6 months.

#### Age differences

Age had no significant influence on results (*P* values > 0.22).

The median age of participants was 62 years, so we examined whether there was a difference between QoL changes in the younger compared to the older half of participants. The baseline values of vitality scores and general health perception scores were significantly different between the two groups (*P* = 0.032 and 0.039, respectively), while there were no differences among the remaining subcategories.

The younger patient group had an immediate and significant improvement in vitality and mental health scores (*P* values at 1 month were 0.020 and 0.033, respectively). General health perception and physical role functioning scores also increased, though not significantly so until the 6 months visit (*P* values at 6 months were 0.0003 and 0.047, respectively).

The only significant changes observed in the older patients were social role functioning and vitality after 3 months (*P* values were 0.0052 and 0.0423, respectively) as well as mental health after 1 month (*P* value 0.023).

No significant changes were present at 6 months in the older patient group.

#### Baseline Ca^2^^+^ differences

Baseline Ca^2+^ also had no significant influence on evolution of QoL scores (*P* values > 0.35).

The median baseline Ca^2+^ value was 1.46 mmol/L. (reference range: 1.18–1.32 mmol/L). Possible differences between the patients with baseline Ca^2+^ above or below the median were examined. There were no significant differences between variables at baseline.

Compared to baseline values, patients with less hypercalcaemia reported significant increases in vitality (*P* = 0.0004), physical role functioning (*P* = 0.004) and mental health perception (*P* = 0.023) which were also present at 6 months.

Patients with more severe preoperative hypercalcaemia reported a significant increase in vitality only when comparing baseline and 6 months scores (*P*= 0.019).

#### Baseline SF-36 values

Incorporating baseline scores in our LMM returned *P* values <0.0001 in every category, that is the baseline value of a subcategory had a significant influence on how the scores changed after successful PTx overall.

The lower-third of each subcategory score at baseline was then calculated separately and values ranged from 25 (physical role functioning) to 75 (physical functioning and social role functioning).

Changes from baseline were evaluated for each subcategory. Given the fixed upper and lower limits of the scoring system, the changes in the group with the lower baseline values were greater. A ceiling effect may have influenced the results, especially for subcategories physical functioning, bodily pain as well as physical, emotional, and social role functioning (>15% of patients scored 100 at baseline in these categories). Among the patients with the lower baseline values, changes to vitality, emotional and social role functioning, and mental health were significant after 1 month. All subcategories except physical role functioning significantly improved after 6 months among the patients with the lower baseline with *P*values ranging from 0.0449 (emotional role functioning) to <0.0001 (vitality and social role functioning).

Patients with a high baseline value reported significantly increased scores in vitality (*P* value 0.031 after 6 months) and mental health (*P* values were 0.007 and 0.030 after 1 and 3 months) though the latter was no longer significant at 6 months.

#### Baseline testing before or after COVID lockdown

It is plausible that the pandemic and ensuing nationwide lockdown could have affected patients’ self-reported QoL. Therefore, we investigated whether there was a difference in baseline values among patients included prior to the nationwide lockdown on 16 March 2020 or after (*n*  = 17 and *n*  = 23, respectively).

Lockdown did not significantly influence any SF-36 category (*P* values > 0.26).

## Discussion

National and international guidelines agree that PTx is the first-choice treatment for PHPT, with the expectation that symptoms will be alleviated and long-term effects of the disease (e.g. osteoporosis, dementia, nephrocalcinosis, etc.) avoided. However, due to earlier disease detection, objective symptoms are fewer and less pronounced. As the likelihood of a PHPT diagnosis increases with age and the subjective symptoms (impaired memory, fatigue, decreased muscle strength, etc.) can be considered part of the expected ageing process, these symptoms may not initially be thought of as indicators of disease. However, there has been some indication that patients experience a quick and lasting improvement in neuropsychiatric symptoms as well as disease-specific symptoms (9, 12, 13, 14, 16, 17, 18). Although few researchers have evaluated changes in QoL as early as 1 month after PTx, our findings concur overall with those of previous publications.

As the symptoms are often vague and non-specific, there may not be much room for perceived improvement. If patients do not feel that the disease affected their QoL prior to treatment, they may be less motivated to undergo surgery. However, PHPT is a slowly progressing disease, so patients may not be aware of the physical or mental effects it has until these are alleviated after successful surgery and they have a new reference point.

With these findings, we hope to clarify which changes in QoL patients with PHPT can expect after PTx – for the group as a whole as well as within their gender, age, or preoperative biochemistry. We found swift and persisting improvements in nearly all SF-36 subcategories with the only exceptions being physical functioning, which improved slowly over time, and pain and emotional role functioning, which did not improve significantly within 6 months. The latter, however, had a median value of 100 at baseline, leaving little room for improvement.

Patients overall expressed a general feeling of improvement at each visit, but the patients included in this study were monitored more closely and thus given the opportunity to speak to a physician more often than the average PHPT patient. This may have added a bias, for which it is impossible to adjust.

We found no significant effect of age or gender on the evolution of QoL subcategories. However, younger participants (< 62 years) appeared to have an overall higher increase in QoL variables. The younger patients likely had fewer concomitant age-related diseases, so after curative treatment for their PHPT, they were free of symptoms, while the older patient population on average may still have been affected by other age-related issues after PHPT cure and therefore did not experience the changes as markedly. Further, the female participants had far more significant increases than their male counterparts with no significant differences in scores at baseline. As these measures are subjective, it is impossible to be certain whether male participants do not, in fact, experience improvement as fast as women, or if they are perhaps less affected by the bias from increased frequency of clinical follow-up.

Unlike the findings of Ejlsmark-Svensson *et al.*, our results showed no effect of preoperative Ca^2+^ on baseline QoL scores or evolution after PTx (18). This could be a true discrepancy or it may be an indicator that the PHPQoL questionnaire used by Ejlsmark-Svensson *et al.* is perhaps more sensitive to specific QoL changes among patients with PHPT than the more generalised SF-36 questionnaire.

Regression towards the mean has undoubtedly been present; however, the QoL changes are very persistent in the measured categories, so the changes seem valid.

The COVID-19 pandemic and nationwide lockdown may well have affected the QoL parameters though no significant differences were found at baseline between patients included before/during the lockdown. A total of 19 patients (17 with successful PTx) were included and underwent baseline testing prior to the nationwide COVID-19 lockdown, while the rest were included during the pandemic. There is no way of knowing the effects the lockdown may have had because the time point for answering the QoL questionnaires and surgery inevitably varied for each patient with respect to the onset and duration of the lockdown.

### Perspectives

Most of the identified SF-36 changes are significant and detectable as early as 1 month after surgery after which further changes are minimal, but we suggest that examining the outcome at 12 months after successful PTx would be interesting in order to evaluate any possible further changes.

The number of participants at 1 and 3 months was low, as well as the number of male participants (14 men compared to 26 women), and it would be interesting to test the current results in a larger cohort to assess whether any minor changes had been missed. This should include evaluation of those patients whose surgical outcome was negative.

In this paper, we examined the self-reported QoL (i.e. a subjective measure), but future studies should also focus on adding objective measures, including changes in muscle strength as well as bone and muscle mass. We intend to follow-up on this aspect in the future.

It is also noteworthy that baseline QoL values can be expected to vary depending on both the duration and severity of PHPT. This will inevitably influence the comparability with future studies of this kind.

## Conclusions

The present data support the notion of a decrease in QoL in a vast number of patients with PHPT irrespective of an only modest increase in calcium and PTH levels and, more importantly, a clinically relevant improvement following successful surgery.

Overall, patients’ QoL scores improved after successful PTx in the categories of vitality, physical functioning, general health perception, physical role functioning, social role functioning, and mental health perception, while no significant changes were identified with regard to emotional role functioning or bodily pain. Emotional role functioning could hardly be expected to increase due to the high proportion of patients scoring 100 at baseline (>60% of patients). The change in bodily pain trended towards significance, but again likely affected by a high proportion of patients with a score of 100 at baseline (28%). There was no difference in reported pain between younger and older patients at baseline (*P* value 0.12), but the visual impression in the Supplementary material (Supplementary Fig. 2, see section on supplementary materials given at the end of this article) suggests that the younger patients may be more aware of PHPT-related pain while older patients may be more accustomed to chronic age-related pain and as a result, they are less affected by PHPT-related pain. The changes with the highest significance scores were vitality and mental health perception.

There was no significant development in QoL scores from 1 month and onwards in the group as a whole.

Subcategorisation showed that changes in vitality and mental health perception can be expected among women under the age of 62. Patients with lower baseline scores had significant increases in almost all categories, although those with a higher baseline score also showed an increase in vitality and mental health perception scores.

The SF-36 QoL questionnaire is a simple and easy method for monitoring changes after PTx. We suggest that guidelines should include symptoms as relative indications for PTx, although our results should be confirmed in a larger cohort. Continuous QoL results throughout the follow-up course could serve as motivation and encouragement for patients who may have misconceptions about the progress that has been made since curative PTx.

## Supplementary Material

Supplementary Material

## Declaration of interest

The authors declare that there is no conflict of interest that could be perceived as prejudicing the impartiality of the research reported.

## Funding

J W C received partial funding from the Scientific Committee of Herlev University Hospital.
