# Supplementary material for: Changes in quality of life 6 months after parathyroidectomy for primary hyperparathyroidism
Source: Endocr Connect. 2022 Feb 23;11(3):e210630. doi: 10.1530/EC-21-0630 (PMC9010815; doi:10.1530/EC-21-0630)

Supplementary material

Figure S1. Evolution of the mean score of each SF-36 subcategory. Red: women. Blue: men.

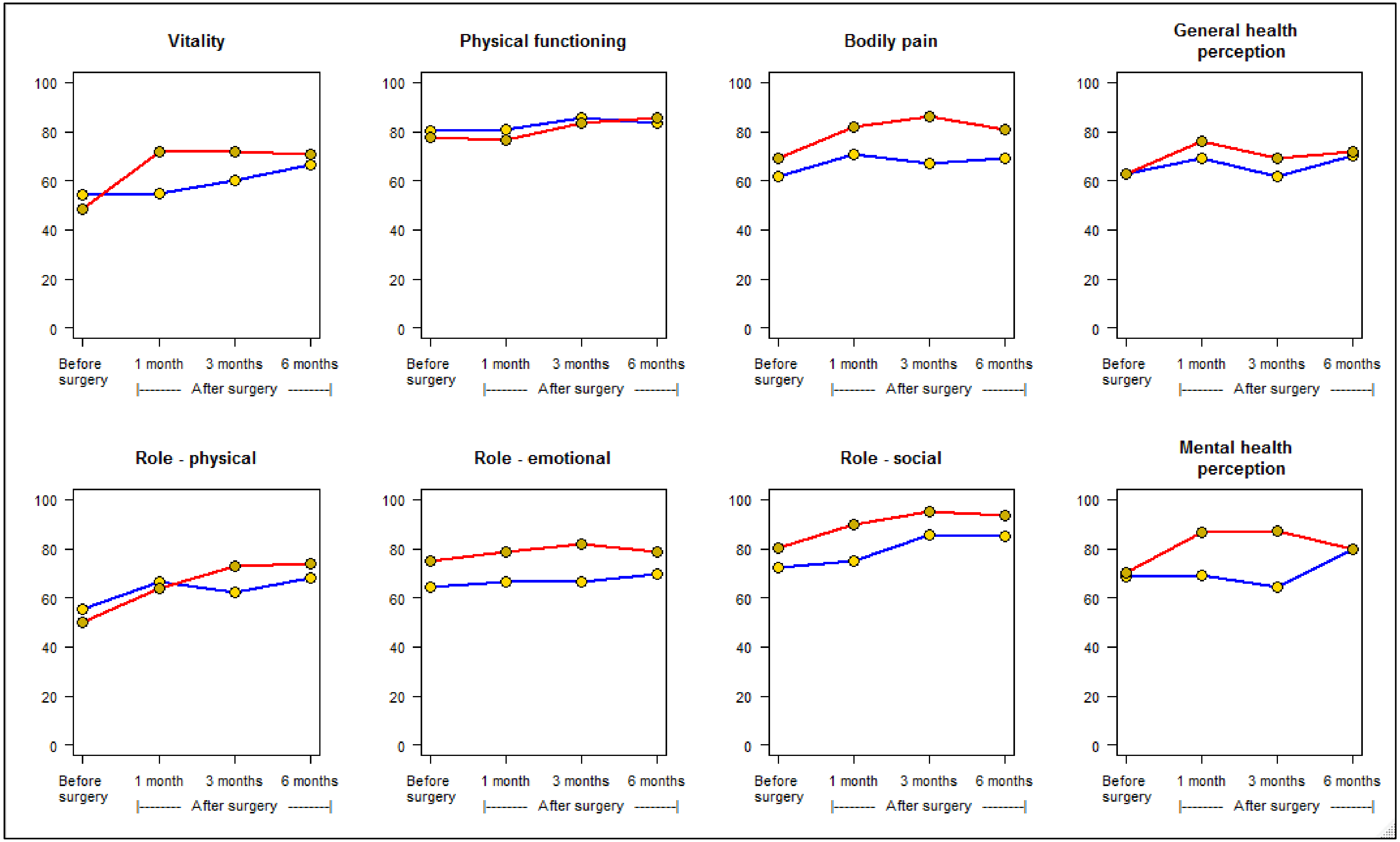

Supplementary material

Figure S2. Evolution of the mean score of each SF-36 subcategory. Red: patients ≥ 62 years at baseline. Blue: patients < 62 years at baseline.

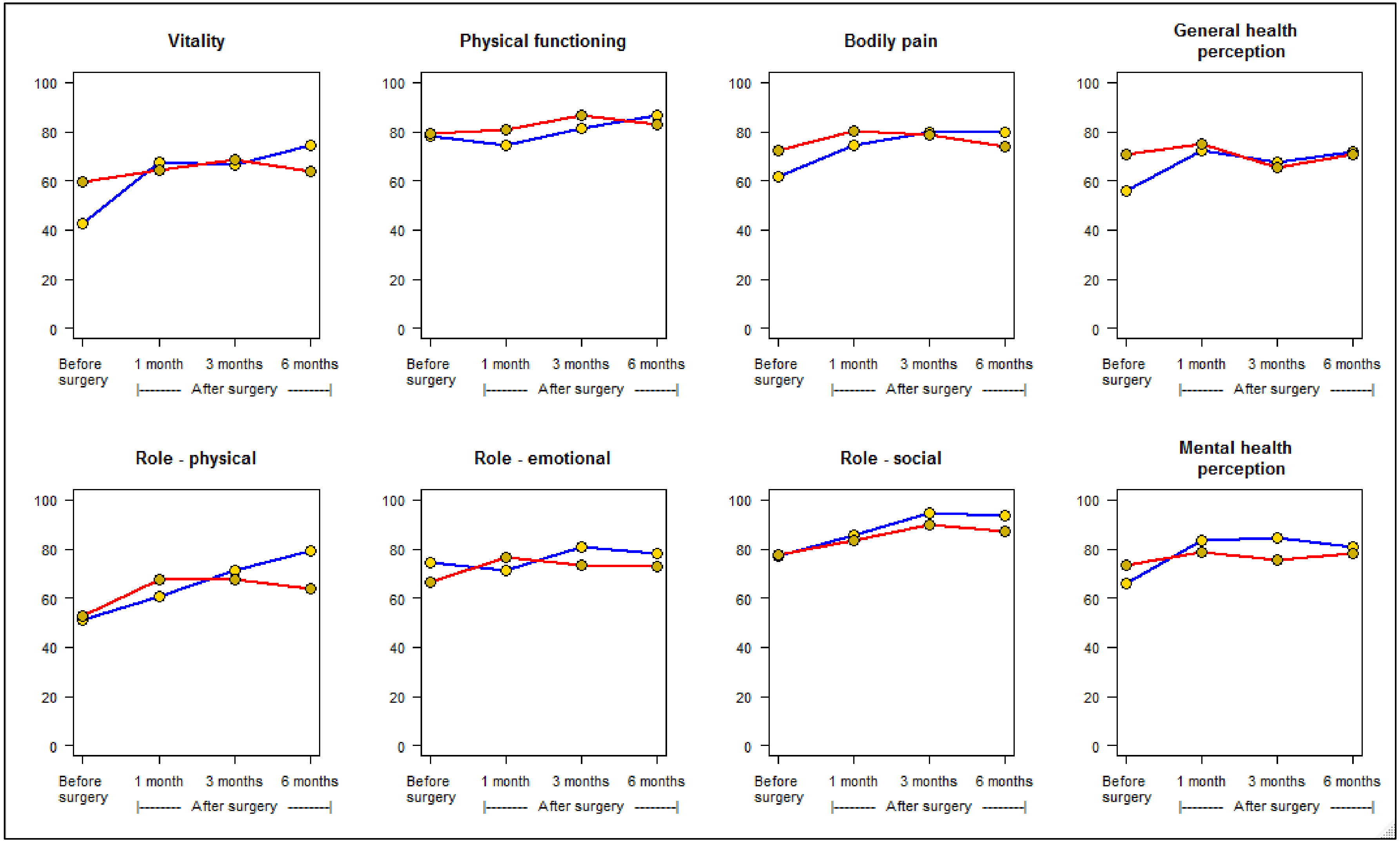

Supplementary material

Figure S3. Evolution of the mean score of each SF-36 subcategory. Red: patients with baseline in the upper 2/3. Blue: patients with baseline in the lower 1/3.

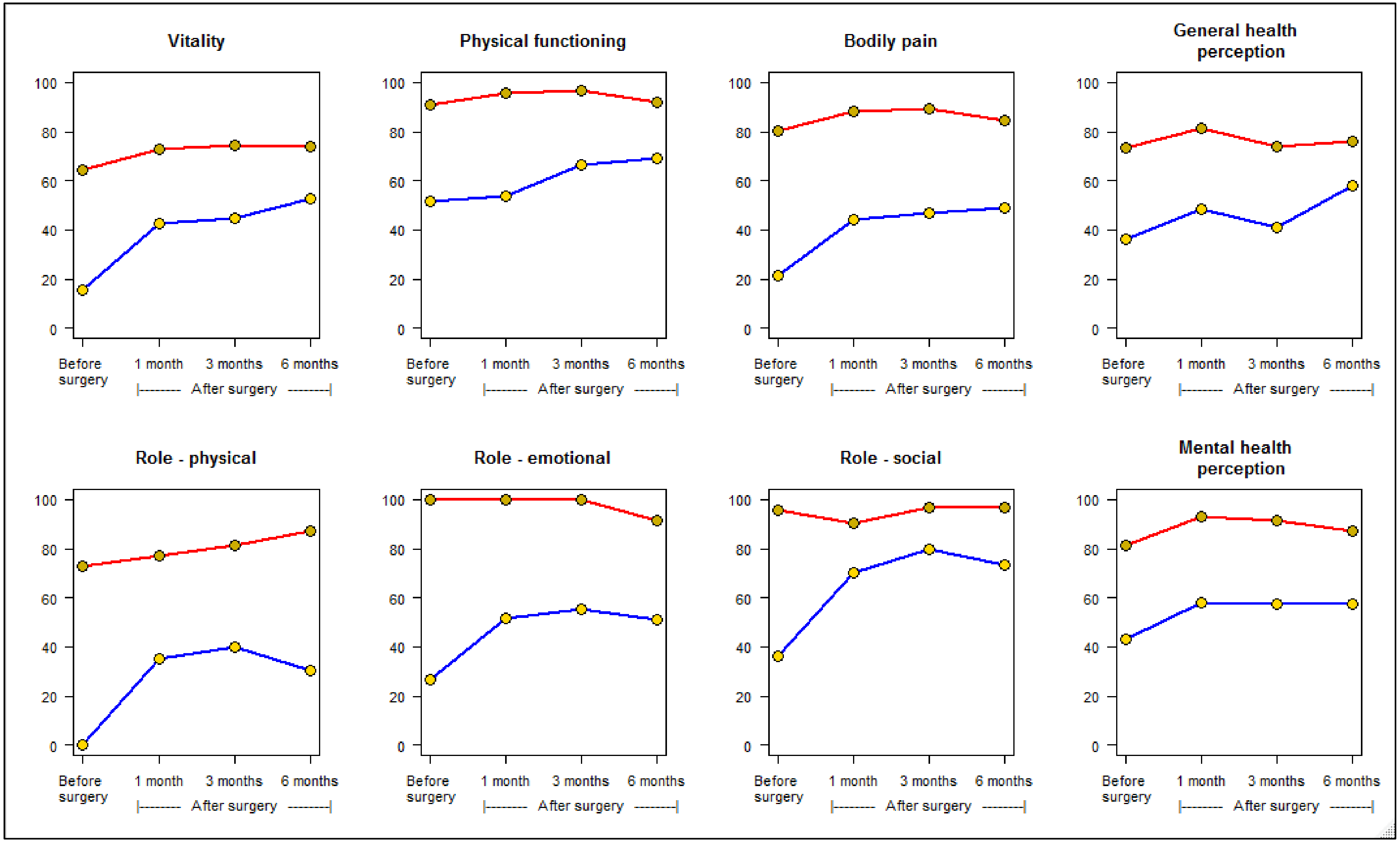

Supplement: Supplementary Material [file supplementary_material.pdf]
